# Proton pump inhibitors may enhance the risk of digestive diseases by regulating intestinal microbiota

**DOI:** 10.3389/fphar.2023.1217306

**Published:** 2023-07-17

**Authors:** Liang Tian, Chongfei Huang, Wenkang Fu, Long Gao, Ningning Mi, Mingzhen Bai, Haidong Ma, Chao Zhang, Yawen Lu, Jinyu Zhao, Xianzhuo Zhang, Ningzu Jiang, Yanyan Lin, Ping Yue, Jinqiu Yuan, Wenbo Meng

**Affiliations:** ^1^ The First Clinical Medical College, Lanzhou University, Lanzhou, Gansu, China; ^2^ Clinical Research Center, Scientific Research Center, The Seventh Affiliated Hospital, Sun Yat-sen University, Shenzhen, Guangdong, China; ^3^ Department of General Surgery, The First Hospital of Lanzhou University, Lanzhou, Gansu, China

**Keywords:** proton pump inhibitors, digestive diseases, microbiota, intestinal flora, *Clostridium difficile*, inflammatory bowel disease, short-chain fatty acids

## Abstract

Proton pump inhibitors (PPIs) are the most used acid-inhibitory drugs, with a wide range of applications in the treatment of various digestive diseases. However, recently, there has been a growing number of digestive complications linked to PPIs, and several studies have indicated that the intestinal flora play an important role in these complications. Therefore, developing a greater understanding of the role of the gut microbiota in PPI-related digestive diseases is essential. Here, we summarize the current research on the correlation between PPI-related digestive disorders and intestinal flora and establish the altered strains and possible pathogenic mechanisms of the different diseases. We aimed to provide a theoretical basis and reference for the future treatment and prevention of PPI-related digestive complications based on the regulation of the intestinal microbiota.

## Introduction

Proton pump inhibitors (PPIs) are currently the most widely used acid-suppressive agents and are the first-line therapy for gastric acid–related disorders, such as peptic ulcer disease, gastroesophageal reflux disease (GERD), and Barrett’s esophagus (Freedberg et al., 2017). PPIs accumulate in parietal cells via circulation throughout the body. The acidic environment surrounding parietal cells prompts the conversion of PPI prodrugs to active metabolites, which then inhibits gastric H^+^, K^+^–ATPase activity in epithelial cells to prevent gastric acid secretion (Sachs et al., 2006; Shin and Kim, 2013). Although PPIs exhibit exceptional acid inhibition effects, various studies have linked PPI use with adverse digestive events, such as small intestinal bacterial overgrowth (SIBO) and *Clostridium difficile* infection. Considering the wide range of PPI applications, these complications warrant further attention.

The human gastrointestinal tract is colonized by 500–1,000 species of bacteria, totaling approximately 10^14^ individuals (Gilbert et al., 2018). Microorganisms that assist the host in physiological and biochemical functions are collectively referred to as intestinal flora (Sender et al., 2016). The intestinal flora is increasingly being highlighted as a key factor in digestive health. Normal intestinal flora can prevent pathogenic microbes from colonizing the digestive tract by depleting surrounding nutrients and producing antimicrobial substances, such as short-chain fatty acids (SCFAs) and bacteriocins (Masanta et al., 2013). Second, the intestinal microbiota produce various metabolites that influence the host gastrointestinal physiological functions (Collins, 2014); one such example is butyrate, which is essential for maintaining the integrity of the colonic epithelium (Jørgensen and Mortensen, 2000). The intestinal flora is also crucial for the maturation of the host mucosal immune system during early life; additionally, the microbiota maintains this regulation throughout life via ongoing interactions with the gastrointestinal mucosa (McDermott and Huffnagle, 2014). Furthermore, the intestinal flora plays an important role in nutrient absorption and energy homeostasis (Flint et al., 2012). Therefore, PPI-induced gut microbiota may significantly affect the corresponding pharmacological action of PPIs.

Numerous studies have shown that PPIs significantly alter the intestinal flora of patients; these changes have been closely associated with PPI-associated digestive complications (Malfertheiner et al., 2017), including functional dyspepsia (FD), SIBO, and *C. difficile*-associated disease (CDAD). Thus, it is of great significance to clarify these PPI-associated changes in the intestinal flora and their corresponding role in different gut disorders. This review aimed to evaluate the association between PPI-dependent alterations in the intestinal flora and resultant digestive diseases. We also discussed the changes in the specific disease-associated strains and their possible mechanisms in related intestinal disorders, with the aim of providing a deeper understanding of PPI-related digestive complications.

## Impact and mechanisms of PPIs on intestinal flora

Normal intestinal flora is primarily composed of *Firmicutes*, *Bacteroidetes*, *Proteobacteria*, and *Actinobacteria*. The dominant bacteria varies between specific sites: gram-positive aerobic bacteria are the dominant species in the duodenum; gram-positive/gram-negative anaerobes and facultative anaerobes are dominant in the ileum; and obligate anaerobes, particularly *Bacteroides*, are the predominant species of the colon (Canny and McCormick, 2008; Molloy et al., 2012). After PPI application, *Planococcaceae*, *Oxalobacteraceae*, and *Sphingomonadaceae* populations have been found to increase in the proximal small intestine (Freedberg et al., 2014); (Shi et al., 2019). The abundance of several taxa, including the orders *Bacillales* (e.g., *Staphylococcaceae*), *Lactobacillales* (e.g., *Enterococcaceae*, *Lactobacillaceae*, and *Streptococcaceae*), and *Actinomycetales* (e.g., *Actinomycetaceae*, and *Micrococcaceae*), were observed to increase in the distal small intestine and blind colon. The abundance of the families *Bifidobacteriaceae*, *Ruminococcaceae*, and *Lachnospiraceae* also decreased following PPI treatment. (Perry et al., 2020). Overall, PPI-induced intestinal bacterial alteration is significant and extensive and may be closely related to many digestive disorders.

**FIGURE 1:**
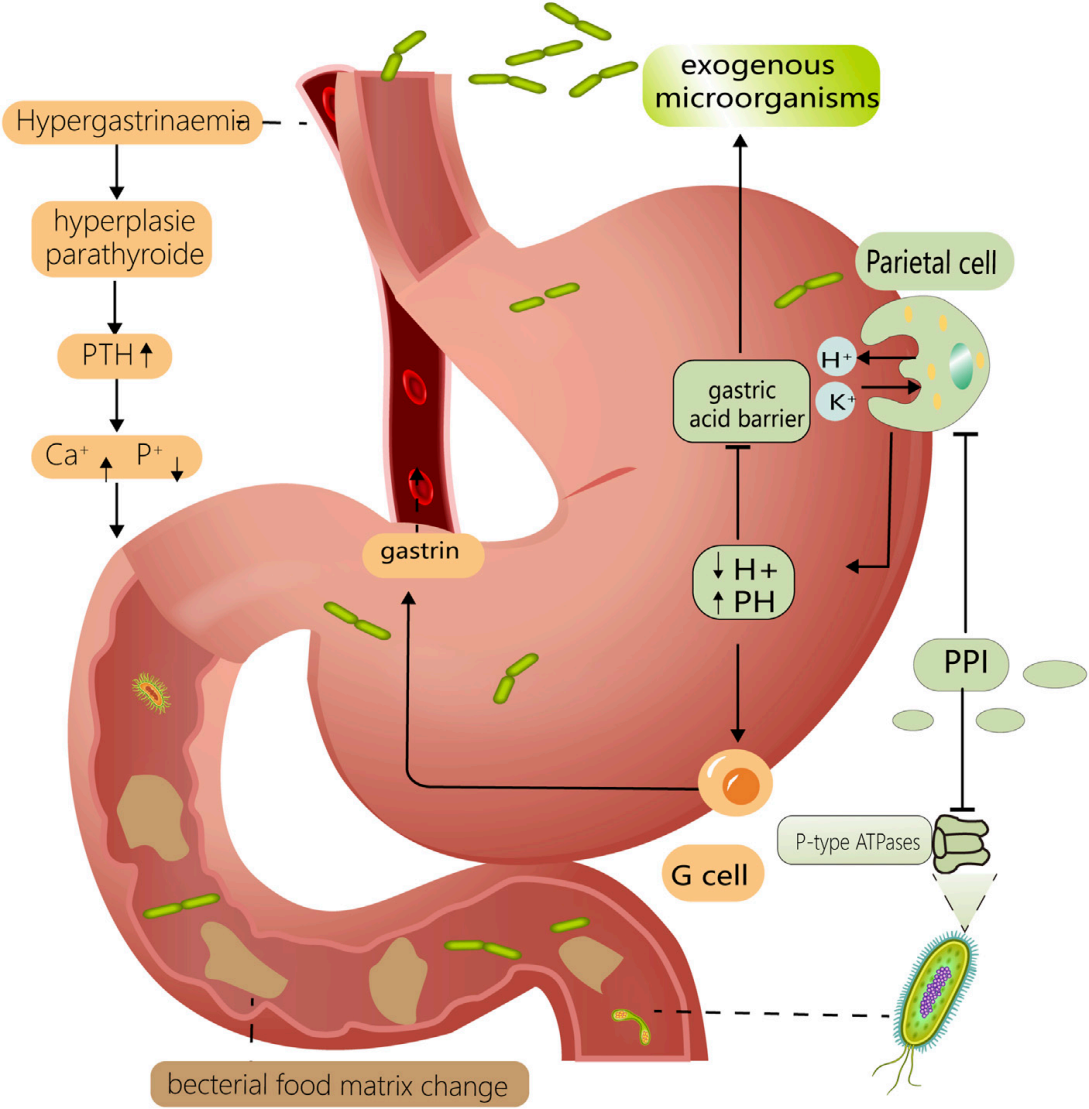
Mechanisms of PPI altering intestinal flora. PPIs reduce gastric acid secretion by inhibiting H⁺/K⁺ ATPase on gastric parietal cells. Lower PH levels compromise the gastric acid barrier, allowing exogenous bacteria to enter the intestine more easily. It also has a prominent effect on microorganisms with specific PH requirements, such as *Helicobacter pylori*. Reduced gastric PH stimulates gastric G-cells to secrete more gastrin, resulting in hypergastrinemia and hyperparathyroidism. This can disrupt the intestinal flora by interfering with the metabolism of calcium and phosphorus in the digestive tract. PPI can also interfere with the P-type ATPase of some gut microorganisms, such as fungi and *Helicobacter pylori*. Above mechanisms work in concert to for PPI to have a considerable effect on the gut flora.

The mechanism by which PPIs affect the intestinal flora can be divided into pH- and non-pH-dependent pathways. First, direct pH modifications alter the environment of the digestive tract; therefore, bacteria with specific pH requirements, such as *H. pylori*, are directly affected (Perry et al., 2020). Thus, pH changes can disrupt the gastric acid barrier, making it easier for exogenous microorganisms to invade the gastrointestinal tract. There are several non-PH-dependent mechanisms by which PPIs affect the intestinal microbiota. First, PPI-induced hormonal changes, such as hypergastrinemia and hyperparathyroidism, affect intestinal osmolality as well as calcium and phosphorus metabolism, which in turn affect the intestinal flora (Yang and Metz, 2010). Second, PPIs influence digestive functions and cause changes in the composition and distribution of digestive tract contents; this may interfere with nutrient absorption functions, thereby altering the quantity or location of the bacterial food matrix and disrupting the intestinal flora (Epstein et al., 2006). Finally, PPIs affect the physiological function of several microbes by directly binding to their non-gastric H^+^/K^+^–ATPases, thereby regulating their corresponding distribution and cell count (Vesper et al., 2009); such affected microbes include fungi, *Helicobacter pylori*, and *Streptococcus pneumoniae* (Melchers et al., 1998; Hoskins et al., 2001). The ways in which PPIs affect the gut microbiota are multifaceted, which may explain their extensive impact (Figure 1).

## PPI-induced digestive system complications

### FD

FD is a digestive disease with numerous complex symptoms that lack obvious pathophysiological features. Nonetheless, *H. pylori* infection, intestinal flora changes, and dysbiosis of the brain–gut bacterial axis may be related to FD (Ford et al., 2020); further, increases in the permeability of the intestine is receiving increasing attention as a potential cause of FD (Tominaga et al., 2007; Vanheel et al., 2014).

Several guidelines recommend PPIs for the treatment of FD to relieve acid reflux and heartburn symptoms or to aid in the eradication of *H. pylori* (Moayyedi et al., 2017). However, a recent study indicated that PPIs may increase intestinal permeability under high psychological stress, which was established based on changes in the adrenocorticotropic hormone–mast cell–vasoactive intestinal peptide–intestinal permeability axis (Takashima et al., 2020). Notably, the results of fecal microbiota transplantation from PPI-administered mice to germ-free mice demonstrated that the intestinal flora is closely related to variations in intestinal permeability.

Further microbiota analysis revealed that a decrease in *Bifidobacterium* abundance was most likely related to PPI-induced FD. *Bifidobacterium* are vital components of the normal intestinal flora and are crucial for the fermentation of carbohydrates into SCFAs (Tsukuda et al., 2021). Butyric acid is an essential SCFA that has a substantial effect on the gastrointestinal tract and is produced by *Bifidobacterium* species. Specifically, butyric acid provides energy for mitochondrial respiration in intestinal epithelial cells (Morrison and Preston, 2016), influences the growth and metabolism of intestinal epithelial cells, and affects the production and distribution of intestinal epithelial connexins (Peng et al., 2009). Furthermore, butyric acid increases smooth muscle excitability in the colon and promotes the release of 5-HT from enteroendocrine cells, both of which are beneficial for intestinal peristalsis and transit (Martin-Gallausiaux et al., 2021). These factors could elucidate the relationship observed between *Bifidobacterium* abundance and permeability variation in the intestinal mucosa. Overall, PPI can affect butyric acid production by reducing the abundance of *Bifidobacterium*, thereby causing changes in intestinal permeability and peristaltic capacity and leading to the onset of FD.

Additionally, the abundances of *Streptococcus*, *Prevotella*, *Veillonella*, and *Actinomyces* are negatively associated with the development of FD (Imhann et al., 2016; Zhong et al., 2017). Further, treatment with *Lactobacillus acidophilus* can reduce the increased intestinal permeability caused by stress in rats (Kang et al., 2022). Overall, the lower abundance of *Streptococcus*, *Prevotella*, and *Lactobacillus* in PPI-treated patients suggests that these strains may also be involved in PPI-induced FD.

Although some studies have found an association between PPI use and increased intestinal permeability, animal research models are not adequate. Additionally, the specific pathogenic mechanisms of the altered strains have not yet been clearly established. Due to the Complexity of FD diagnosis and treatment, there remains a scarcity of high-quality clinical studies pertaining to FD, which further restricts research into the exact role of PPIs in FD. We advise patients with FD to exercise caution when using PPIs and recommend discontinuation of PPI mediation once clinical symptoms are alleviated; doing so may produce significant benefits for these patients.

### Lower gastrointestinal bleeding

PPIs are widely used to prevent gastrointestinal bleeding induced by NSAIDs, aspirin, and antiplatelet medications. However, recent clinical trials have demonstrated that the positive effect of these PPIs is primarily restricted to the upper gastrointestinal tract; in fact, PPIs have even been associated with bleeding in the lower gastrointestinal tract (Watanabe et al., 2008; Nagata et al., 2015; Washio et al., 2016).

In 1996, Uejima et al. (1996) established that the intestinal flora, especially gram-positive bacteria, such as *Escherichia coli*, play an important role in NSAID-induced gastrointestinal injury. Nadatani et al. (2019) determined that PPIs enhance the risk of indomethacin-induced small intestinal injury; further, similar small intestinal injuries were observed following the transplantation of feces from PPI-treated mice into germ-free mice. Additional microbiota analysis indicated that the primary cause of these small intestinal injuries may be a corresponding decrease in the abundance of *Lactobacillus johnsonii*; corresponding, the trend of damage aggravation was significantly inhibited following *L. johnsonii* supplementation (Nadatani et al., 2019). The protective effect of *L. johnsonii* may be associated with its production of lactic acid (Watanabe et al., 2009). NSAID-induced intestinal injury is primarily mediated by activation of the lipopolysaccharide/Toll-like receptor 4 signaling pathway, which, in turn, activates NF-κB to promote tumor necrosis factor-α (TNF-α) release from monocytes cells. Both *in vivo* and *in vitro* experiments have demonstrated that lactic acid, produced by *L. johnsonii*, can inhibit the phosphorylation of I-κb to reduce its degradation; then, I-κb can bind to NF-κB to inhibit its activation and, thus, reduce NSAID-induced intestinal injury (Watanabe et al., 2009).

Another study also confirmed the importance of intestinal flora. Sequencing analysis revealed a decrease in the abundance of the phylum *Actinomycetes*, particularly *Bifidobacterium*, in the jejunum of individuals with gastrointestinal bleeding; nonetheless, intestinal damage was alleviated by *Bifidobacterium* supplementation (Wallace et al., 2011). Recent research has established that the common probiotic *Bifidobacterium* has many effects on the gut. For example, species of this genus optimize the structure of the intestinal flora, antagonize the colonization of *E. coli*, inhibit the activation of NF-κB (Bermudez-Brito et al., 2012), enhance the function of the intestinal mucus layer and cupped cells, and increase the production of mucin; ultimately, these functions may be associated with its corresponding ability to reduce gastrointestinal bleeding (Engevik et al., 2019). However, the specific underlying mechanisms of *Bifidobacterium* require further clarification. Alternatively, a clinical study reported that after patients were administered *Lactobacillus casei* for 3 months, gastrointestinal bleeding symptoms were partly relieved. However, the study design of this trial was incomplete, lacking a placebo control group and utilizing a small sample size (Endo et al., 2011).

Existing studies have documented that the intestinal flora is closely linked to PPI-induced lower gastrointestinal bleeding; however, the majority of these studies were conducted on animal models, the hierarchy of evidence was low, and the results of different studies lacked consistency with each other. The normal intestinal flora is maintained in a dynamic equilibrium based on the interaction between various microbial strains; this internal effect contributes to the complexity of the intestinal flora, which may explain the differences observed in different studies. Recent studies have focused more on the role of specific strains rather than on the overall effect of the microbiota, which also limits further understanding of the association between the intestinal flora and gastrointestinal bleeding. Ultimately, advanced sequencing technologies and multidisciplinary approaches may provide a better understanding of these interactions.

### SIBO

SIBO is a disease in which the number of bacteria in the small intestine increases abnormally, resulting in the production of large amounts of hydrogen, methane, and SCFAs, thereby causing symptoms, such as bloating and diarrhea (Erdogan et al., 2015). The clinical diagnostic criteria for SIBO are not uniform, and small intestinal aspirate content culture alongside methane (CH_4_) and hydrogen (H_2_) exhalation testing are commonly used to further clarify the diagnosis (Pimentel et al., 2020).

In 1994, Fried indicated that PPI and SIBO may be related (Fried et al., 1994). Subsequently, numerous studies have been conducted to explore this issue; however, the corresponding results have been inconsistent. An early meta-analysis found no association between PPI use and SIBO prevalence (Lo and Chan, 2013). However, subsequent meta-analysis in 2018 indicated that PPI use moderately increased SIBO risk; nonetheless, subgroup analysis based on different diagnostic methods indicated a correlation between PPI use and SIBO when assessed using small intestine aspirate culture or the glucose hydrogen breath test but not when using the lactulose hydrogen breath test (Su et al., 2018). In a recent clinical trial that diagnosed SIBO using both small intestine aspirate contents and stool sample cultures, the small intestinal flora of PPI-treated and non-PPI-treated individuals was not significantly different (Weitsman et al., 2022).

This substantial disparity between studies can be attributed to various factors. First, SIBO is linked to the development of GERD (Schatz et al., 2015); this correlation may be attributed to the additional intestinal gas in patients with SIBO, causing an increase in intra-abdominal pressure, which could lead to a large reflux of gastric contents into the esophagus (Takakura and Pimentel, 2020). Nonetheless, GLP-1 and YY peptides, produced by intestinal fermentation, can delay gastric emptying and lead to the transient relaxation of the lower esophageal sphincter (Urita et al., 2006); therefore, PPI, as a guideline-recommended drug for GERD, indirectly increases the occurrence of SIBO in patients with GERD. Secondly, PPI use is more prevalent in older people, which makes the participants in some studies older. Overall, older people have a higher incidence of SIBO due to poorer gastrointestinal function, which could lead to an indirect association between PPI and SIBO (Rao and Bhagatwala, 2019). Finally, different studies utilize differing diagnostic methods. At present, aspirated intestinal content culture is the gold standard for the diagnosis of SIBO, with a number of colonies >1 × 10^5^ colony forming units/mL being diagnosed as SIBO. However, this method is invasive and challenging (Adike and DiBaise, 2018). Although methane and hydrogen breath tests are commonly used, they lack precise operating guidelines and diagnostic standards. Furthermore, the prevalence rates obtained for the same group of patients using the breath test and small intestinal content culture differed significantly (Shah et al., 2020); therefore, diagnostic methods may be the greatest limitation in exploring the link between PPIs and SIBO.

Numerous studies have focused on the association between PPIs and SIBO; however, most clinical studies and meta-analyses have not controlled for various confounding factors, such as dose, duration, and type of PPI exposure, the inclusion and exclusion criteria were also insufficiently stringent across these studies. Thus, the relationship between PPIs and SIBO ultimately remains unclear. New sequencing techniques or metabolite analyses of the intestinal flora may be of high significance for SIBO diagnosis; this could be used to effectively avoid the aforementioned issues, thereby producing high quality clinical studies to address this problem.

### CDAD

In recent years, CDAD has gained increasing attention as a potential PPI-induced gastrointestinal complication. *Clostridium difficile* is a gram-positive anaerobic bacterium that is carried by approximately 3% of healthy adults; however, this proportion can reach 50% in long-term hospitalized patients (Poutanen and Simor, 2004).

*Clostridium difficile* most often takes the form of dormant spores (Kelly and LaMont, 1998); however, antibiotics, particularly fluoroquinolones, can promote spore conversion to the active trophic form, which can ultimately induce CDAD (Pépin et al., 2005). Some studies have demonstrated that PPIs can also increase the risk of developing CDAD (Dial et al., 2004; Akhtar and Shaheen, 2007; Hong et al., 2019); a recent retrospective study demonstrated that this PPI-mediated effect can reach up to 7.15-fold (Hung et al., 2021)and may be more pronounced when combined with antibiotics (Archimandritis et al., 1998).

Several potential mechanisms have been established for PPI-associated CDAD. First, PPIs regulate the gene expression levels of *C. difficile*–related toxins directly. When *C. difficile* is exposed to PPIs, the maximum expression levels of toxin-associated genes can reach approximately 120-fold the corresponding normal levels (Stewart and Hegarty, 2013). Among them, toxin A primarily affects the intestinal epithelium and can cause cytoskeletal actin disassembly via glycosylation of small GTPases in epithelial cells; this can eventually lead to cell death and increase colonic epithelial permeability, which induces the occurrence of CDAD (Wetzel and McBride, 2020). Second, elevated pH levels caused by PPIs accelerated the transition of *C. difficile* spores to the viable trophic form (Nerandzic et al., 2009). Third, under normal conditions, normal salivary nitrite can mix with gastric acid to create nitrogen oxides that can kill *C. difficile*; however, this effect is greatly diminished by the pH increase caused by PPI treatment (Cunningham et al., 2014). Finally, PPI treatment disrupts normal intestinal flora; the occurrence of CDAD is closely correlated with the overall change in intestinal microbiota induced by PPI (Clooney et al., 2016). The efficacy of fecal transplantation for CDAD also confirms the significance of normal intestinal flora in this disease (Broecker et al., 2016). Therefore, several recent clinical studies have supported the use of probiotics, such as *Lactobacillus* and yeast, for the treatment and prevention of CDAD (Goldenberg et al., 2017). Collectively, these results confirm the correlation between PPI treatment and CDAD.

Although many studies have indicated an association between PPI use and CDAD, most were retrospective studies with significant heterogeneity and relatively low quality of evidence. Overall, it is necessary to exercise caution when prescribing PPIs to patients with CDAD risk factors, such as long-term hospitalization and a history of CDAD; additionally, probiotics should be considered as a novel strategy for the prevention of CDAD.

### Gastrointestinal infectious diseases

PPI users are more likely to develop bacterial gastroenteritis, which is primarily caused by *Salmonella* and *Campylobacter* (García Rodríguez et al., 2007; Doorduyn et al., 2010; Wu et al., 2014). Several mechanisms have been established for PPI-associated bacterial gastroenteritis. First, PPIs disrupt the gastric acid barrier, allowing exogenous bacteria, particularly acid-resistant bacteria, to enter the gastrointestinal tract easily (Martinsen et al., 2019). According to *in vitro* tests, most exogenous bacteria, including *Vibrio cholerae* and *Campylobacter jejuni*, are disturbed at a pH of 3.0; however, *Salmonella* and *Campylobacter* can still persist under these conditions and can rapidly multiply due to their shortened incubation period (Foley et al., 2013). Second, normal intestinal flora can antagonize the colonization of pathogenic bacteria through the production of antimicrobial substances, such as organic acids, bacteriocins, and competing nutrients (Zhao et al., 2018); however, PPI treatment disrupts the balance of intestinal flora, leading to a reduction in the normal antagonistic effect against exogenous bacteria. Further, this altered gut microbiota has been reported to instead facilitate the exposure of intestinal epithelial cells to pathogenic bacteria (Freedberg et al., 2014) and may even provide a protective environment for these ingested pathogens (Masanta et al., 2013). Finally, PPIs inhibit lysosomal acidification by suppressing the neutrophil vacuolar-type H^+^ ATPase (V-ATPase), which ultimately reduces neutrophil antibacterial activity (Song et al., 2017).

Despite numerous clinical studies supporting the link between PPI and bacterial gastroenteritis, most prior research designs are imperfect. Some studies have suggested that excluding participants who had gastroenteritis prior to the first use of PPIs or correcting for confounding factors, such as susceptibility and time dependence of bacterial gastrointestinal infections, can remarkably reduce the association between PPI treatment and bacterial gastroenteritis (Garcia Rodríguez and Ruigómez, 1997; Hassing et al., 2016).

Diseases caused by fungal infections of the digestive system have also been linked to the use of PPIs, particularly *Candida* infections (Mottaghi et al., 2021). In addition to the factors discussed above, the PPI-induced reduction in *Lactobacillus* can also prompt fungal infections, the presence of *Lactobacillus* typically inhibits the growth of fungi, such as *Pseudomonas*, via the production of lactic acid (Wang et al., 2004).

PPIs have also been shown to be associated with intestinal infections caused by multidrug-resistant microorganisms. A recent meta-analysis demonstrated that PPI use increased infection rate by 75% (Willems et al., 2020), including infections by multidrug-resistant *Enterobacteriaceae* and vancomycin-resistant *Enterococcus*. The potential mechanisms for this increased infection rate include the PPI-dependent disruption of the gastric acid barrier and microecological balance (Nakamura et al., 2019).

Overall, the current literature primarily supports the link between PPI treatment and bacterial gastroenteritis; however, most results were obtained via single-center observational and retrospective studies and lack information on confounding factors, such as duration of PPI exposure, antibiotic intake, and dietary patterns. More rigorous clinical studies and higher-quality evidence are needed to establish the strength and cause-and-effect relationship of this correlation. Overall, it is also crucial to consider the risk of enteric infection when prescribing PPIs.

### Cholelithiasis and biliary system diseases

Cholelithiasis, or gallstone formation, is one of the most prevalent disorders of the digestive system, with numerous causative factors, including family history, female sex, and lack of physical activity. PPI has been demonstrated to increase the occurrence of gallstone disease (Fukuba et al., 2017; Yang et al., 2021); further, intestinal flora also play a critical role in gallstone formation because of its corresponding influence on bile acid and cholesterol metabolism (Wang et al., 2020).

There are several known pathogenic mechanisms of PPI in cholelithiasis. First, the intestinal flora composition of mice chronically exposed to PPIs is comparable to that of mice fed with high-fat diet (Yang et al., 2020); importantly, a high-fat diet has been clearly identified as a risk factor for gallstone formation. Second, PPI use increases the abundance of producing bile salt hydrolase (BSH) bacteria, such as *Lactobacillus* and *Clostridium*. These bacteria play an important role in bile acid metabolism via the depolymerization of glycine or taurine from conjugated bile acid sterol cores; then, primary bile acids are metabolized to secondary bile acids by 7α-dehydroxylase (Foley et al., 2019). Elevated BSH activity causes an increase in secondary bile acids in the intestine, and some secondary bile acids, especially deoxycholic acid, can promote gallstone formation via enterohepatic circulation (Wang et al., 2020). Third, in the intestine, 7α-dehydroxylase is mainly produced by *Clostridium* and *Eubacterium*, which also increases their abundance and can contribute to gallstone formation by increasing secondary bile acid production (Jia et al., 2018). Finally, PPIs decrease cholecystokinin release by delaying gastric emptying, thereby attenuating gallbladder motility and delaying gallbladder emptying (Cahan et al., 2006). Long-term PPI use may also promote bile duct epithelial proliferation and bile duct epithelial micropapillary growth, resulting in focal bile duct stenosis and obstruction, which may also be associated with gallstone formation (Yang et al., 2020).

Although current clinical studies have demonstrated a correlation between PPIs and cholelithiasis, the specific effect of PPI-induced changes in the intestinal flora on cholelithiasis remains unclear. PPIs also cause the reduction of other bacteria that produce BSH, such as *Bifidobacterium*; however, studies on the direct impact of PPI on the activity of BSH and 7α-dehydroxylase are limited (Hu et al., 2022).

The relationship between PPIs and infectious diseases of the biliary system has been discussed in several clinical studies (Chuang et al., 2019; Min et al., 2019). The biliary system typically maintains a sterile environment with bile acids (D'Aldebert et al., 2009); however, a recent study proposes that there is a clear transfer of intestinal microorganisms to the biliary tract in patients with biliary tract diseases (An et al., 2022). Additionally, animal models of SIBO revealed that intestinal flora and metabolites can affect bile acid metabolism and cause biliary disease (Almeida et al., 2013); overall, this establishes a correlation between biliary system disease and the intestinal microbiota. In recent years, correlational studies of PPI and bile duct cancer have also been reported (Kamal et al., 2021); however, we believe that this effect is more likely indirectly caused by PPI-induced cholelithiasis and biliary tract infections.

Many studies on the effects of PPIs on cholelithiasis and biliary system diseases are retrospective analyses based on large-scale epidemiological databases that are prone to residual confounding factors. These confounding factors are derived from the limitations in the research methodology and are difficult to adjust. Therefore, a higher level of evidence, such as results from randomized controlled trials, is needed to clarify these findings. Due to the connection between the hepatobiliary system and the small intestine, alongside the important role of the intestinal flora in bile acid metabolism, the use of PPIs can potentially impact the biliary tract through the intestinal flora. Therefore, long-term PPIs users should remain vigilant about the health of their hepatobiliary system.

### Inflammatory bowel disease (IBD)

IBD is a chronic inflammatory disease of the intestine that includes ulcerative colitis and Crohn’s disease. The etiology of IBD is diverse, with genetic factors, environmental factors, intestinal flora disruption, and immune imbalance being identified as potentially risk factors (Zhang and Li, 2014). In particular, the intestinal flora in a transgenic mouse model of IBD showed an intestinal inflammatory response only when intestinal microbes were present, emphasizing the importance of the intestinal microbiota in IBD (Lloyd-Price et al., 2019). In recent years, several large population cohort studies have indicated a link between PPI use and the occurrence of IBD (Allin and Moayyedi, 2021; Lo et al., 2020; Xia et al., 2021) Further microbiota analysis have indicated a strong similarity between changes in the intestinal flora of patients with IBD and PPI participants, suggesting that the gut microbiota could play a significant role in PPI-induced IBD (Issa et al., 2007).

There are multiple potential mechanisms by which PPI use can induce IBD. First, PPI use increases the risk of intestinal bacterial infections, which are considered typical initiating events of IBD (Rivera-Chávez et al., 2016). Second, PPI treatment promotes the occurrence of CDAD, which is a common cause of IBD (Sokol et al., 2008). Third, the abundance of the protective bacteria *Faecalibacterium prausnitzii* is reduced in the intestine. Notably, *in vitro* and *in vivo* studies have shown that *F. prausnitzii* exhibits an anti-inflammatory effect; specifically, its metabolites possess the ability to inhibit NF-B activation and IL-8 secretion, which are strongly linked to intestinal inflammation. Thus, reducing the abundance of *F. prausnitzii* may result in a high risk of IBD relapse and a reduced duration of remission (Sokol et al., 2008; Rajca et al., 2014). Finally, The abundance of *Lactobacillus reuteri* is reduced following PPI use. *L. reuteri* has the ability to convert the histidine present in food to histamine. Then, histamine increases cAMP levels by activating histamine receptors (H2); next, cAMP inhibits downstream MEK/ERK–MAPK signaling via protein kinase A and suppresses the production of TNF-α, a key driving factor for chronic inflammation in IBD. Therefore, a decrease in *Lactobacillus royi* may also contribute to IBD development (Thomas et al., 2012). Furthermore, the relationship between bile acids and IBD has recently received significant attention (Lloyd-Price et al., 2019); in particular, deoxycholic acid has been observed at significantly low levels in patients with IBD but dramatically increases after treatment (Ding et al., 2020). Therefore, PPI-induced bile acid alterations may also be an influencing factor in PPI-associated IBD.

**FIGURE 2:**
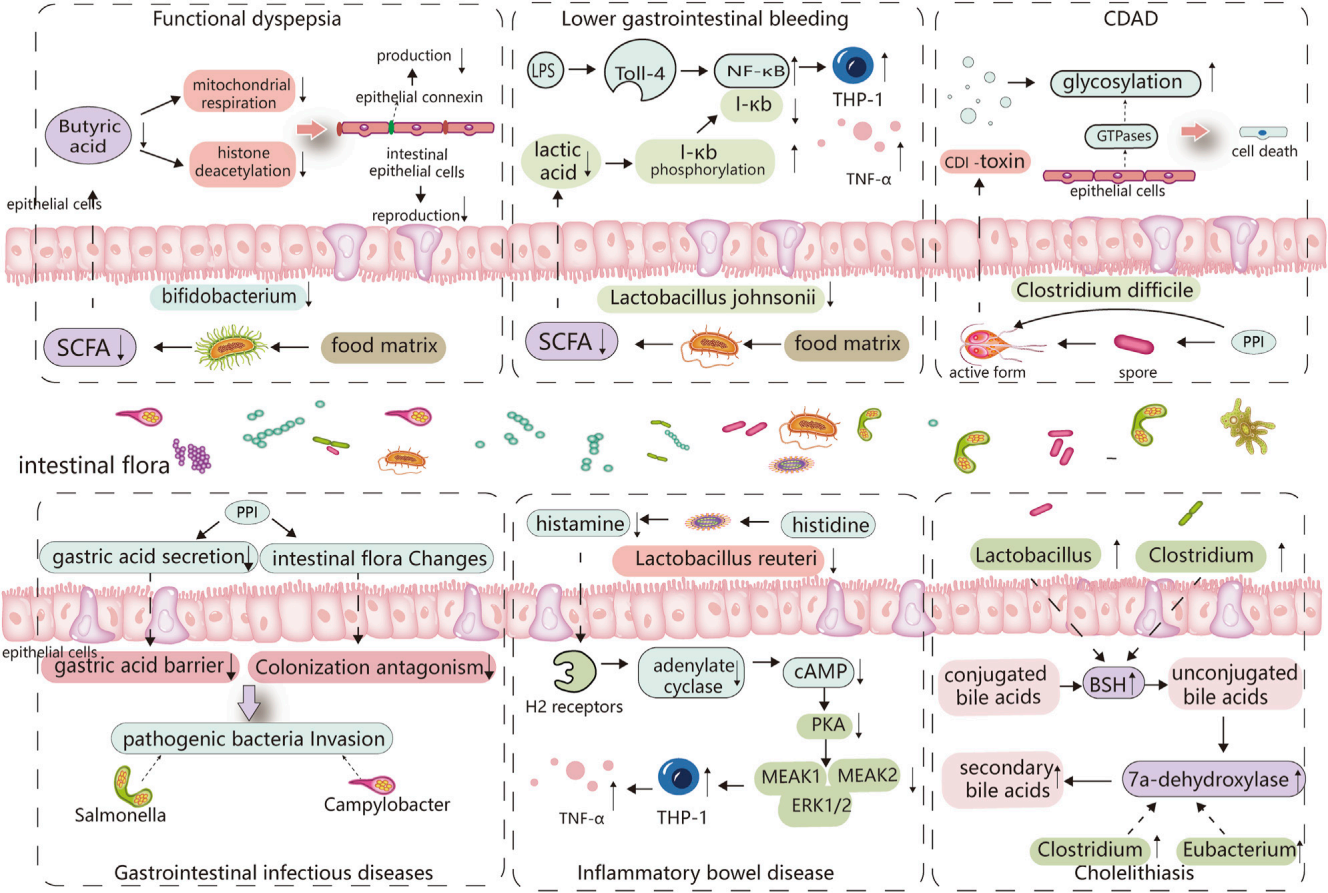
Major altered bacterial strains and possible pathogenesis in different PPI-induced digestive diseases. The altered gut microbiota caused by PPIs and its digestive side effects are inextricably linked, with certain strains of bacteria playing an important role. Reduced *Bifidobacterium* abundance limits the production of butyric acid, which is linked to the reproduction of intestinal epithelial cells and the synthesis of epithelial connexin. Butyric acid deficiency increases epithelial permeability and induces functional dyspepsia. *Lactobacillus johnsonii* is linked to the production of lactic acid, which can inhibit the phosphorylated degradation of I-κb and promote its binding to NF-κB, thereby inhibiting TNF-α release from monocytes cells, When the abundance of *Lactobacillus johnsonii* decreases, THP-1 cells produce more TNF-, which is linked to PPI-induced lower gastrointestinal bleeding. PPI directly promotes *Clostridium difficile* toxin expression, and elevated pH also stimulates *Clostridium difficile* spore germination, which is associated with C. difficile-associated diseases. PPI disrupts the gastric acid barrier and normal intestinal flora, allowing exogenous pathogenic bacteria to invade and cause gastrointestinal infectious diseases. *Lactobacillus reuteri* can convert histidine to histamine and activates H2 receptors, cause adenylate cyclase produce more cAMP, which through PKA restrains downstream MEK/ERK-MAPK signaling, inhibiting the production of TNF-α, thus delaying the incidence of IBD. So, *Lactobacillus reuteri* reduction promotes the occurrence of IBD. PPI increases the abundance of bacteria that produce 7a-dehydroxylase and bile salt hydrolase in the intestine, comprises *Eubacterium*, *Clostridium*, and *Lactobacillus*, thereby cause an increase in the synthesis of secondary bile acids, especially deoxycholic acid (LDA). These bile acids act as a lithogenic core and promote the development of cholelithiasis.

Numerous clinical studies have verified a correlation between PPIs and IBD. However, further cohort studies are required to strengthen these findings; additionally, more basic research is required to determine the primary pathophysiological mechanisms and the role of intestinal flora in PPI-induced IBD. In recent years, regular administration of probiotics, such as *Bifidobacterium* and *Lactobacillus*, has also been considered a potential method for preventing IBD (Manzoor et al., 2022), which further indicates the importance of the intestinal flora in IBD (Figure 2; Table 1).

**TABLE 1:** Major changed gut bacteria affected by PPI in different diseases.

Disease	Changed flora	References
Functional dyspepsia	*Bifidobacterium*	Takashima, S
	*Streptococcus*, *Prevotella Veillonella*, *Actinomyces*	Imhann, F
	*Lactobacillus acidophilus*	Kang, Y
Lower gastrointestinal Bleeding	*Lactobacillus johnsonii*	Nadatani Y
	actinomycetes	Wallace, J.L
	*Lactobacillus casei*	Endo, H
*Clostridium difficile* associated diarrhea	*Clostridium difficile*	Nerandzic, M.M
	*Bacteroides*, *Firmicutes*	Clooney, A.G
	*Lactobacillus*, yeast	Goldenberg, J.Z
Gastrointestinal infectious Diseases	*Salmonella*	Wu, H.H
	*campylobacter*	Doorduyn, Y
	*Candida*	Mottaghi, B
	*Vancomyc inresistant enterococci*	Willems, R.P.J
	*Escherichia coli*	Nakamura, A
Cholelithiasis	*Lactobacillus*, *Clostridium*	Foley, M.H
	*Eubacterium*	Jia, W
Inflammatory bowel disease	*Faecalibacterium prausnitzii*	Sokol, H
	*Lactobacillus reuteri*	Thomas, C.M

## Discussion

**FIGURE 3:**
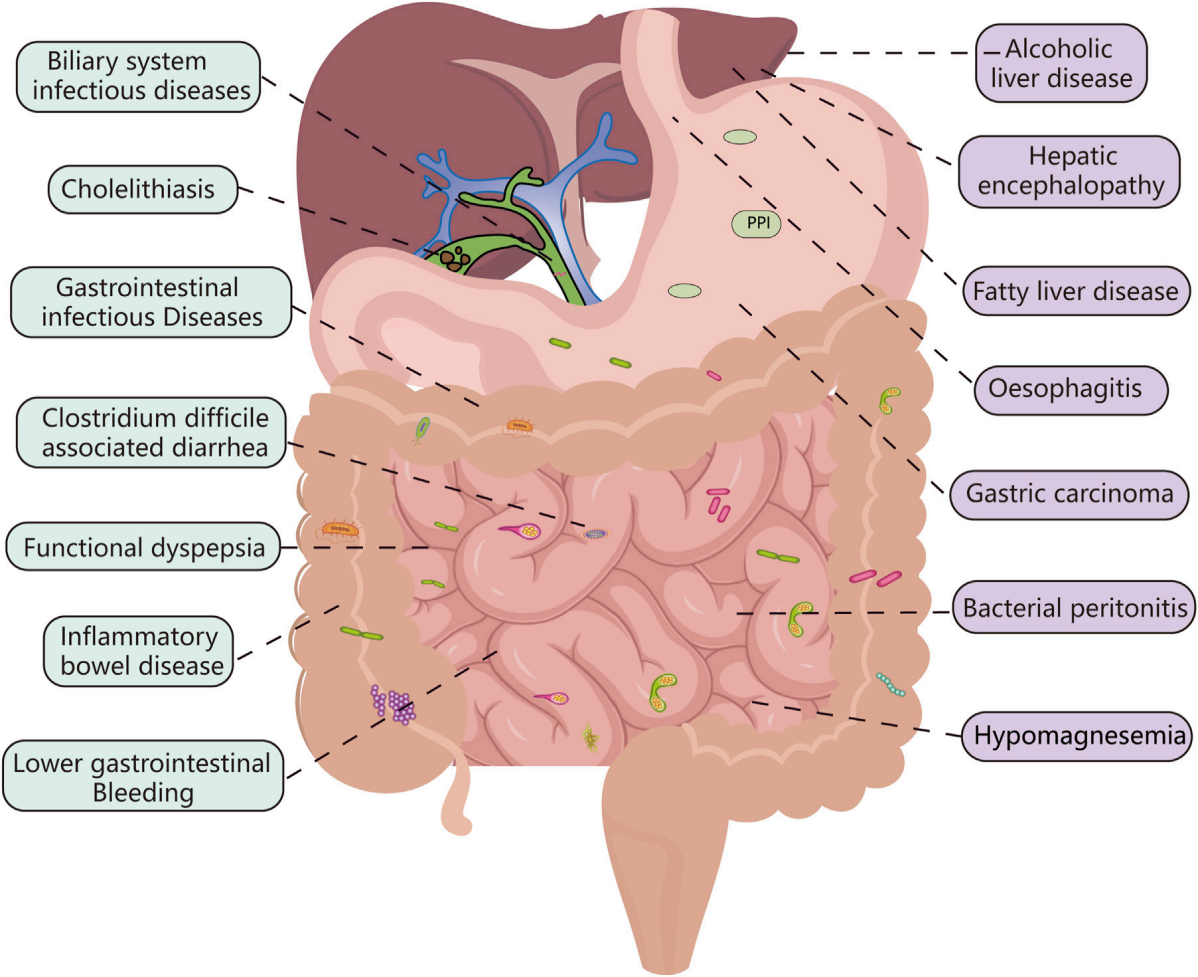
PPI-related digestive diseases: the connection between the green-labeled diseases and the intestinal flora is more obvious, while the purple-labeled diseases require more research.

Over the past decade, there has been an overwhelming amount of literature on the adverse digestive effects of PPI use; further, several studies have highlighted the significant role of the intestinal flora in the relationship between PPI and the digestive system. However, there is a lack of comprehensive literature regarding the role of the gut microbiota in PPI-related digestive complications. In this review, we evaluated the overall changes to the intestinal flora alongside variations to the specific strains within the microbiota and established their association with PPI-related digestive disorders whilst detailing all possible mechanisms of action involved in this relationship (Figure 3).

The PPI-mediated alteration of bacterial metabolites, such as SCFAs, lactic acid and histamine, is a crucial mechanism in the development of certain digestive complications. For example, SCFAs, which are mainly produced by *Bifidobacterium* and *Lactobacillus* species, are related to intestinal permeability, immunity, and hormones (Dalile et al., 2019). Among these SCFAs, butyrate can directly affect other intestinal bacteria via cross-feeding (Koh et al., 2016). (Huang et al., 2020). This may explain the association between PPIs and FD, intestinal infectious diseases, and CDAD. Emerging evidence suggests that SCFAs play critical roles in various digestive diseases. However, the precise effect of PPI on SCFA production is unknown; therefore, further research into the SCFAs produced by PPI-associated bacteria may be helpful in understanding the relationship between intestinal flora and PPI-related digestive complications. Moreover, PPIs can disrupt the gastric acid barrier and intestinal microecology and cause exogenous bacterial invasion, which can lead to infectious diseases of the gastrointestinal and biliary tracts and SIBO. PPIs can also directly affect pathogenic bacteria, such as *C. difficile*, which results in CDAD; however, this is a rare phenomenon.

The intestinal flora also plays an important role in other PPI-mediated diseases. Disruption of the gut microbiota affects the fermentation of the intestinal contents, resulting in hypomagnesemia (Thongon and Krishnamra, 2012; William and Danziger, 2016). Additionally, an increase in the abundances of *Proteobacteria* and *Streptococcus* species has been linked to the development of rheumatoid arthritis (Imhann et al., 2016; Kim et al., 2021). Other related diseases include gastric cancer (Lofgren et al., 2011), bacterial peritonitis (Preto-Zamperlini et al., 2014; Wellhöner et al., 2019), hepatic encephalopathy (Bian et al., 2017; Tsai et al., 2017), and alcoholic fatty liver (Llorente et al., 2017; Pyo et al., 2021).

Specifying the advantages and disadvantages of PPI use for each individual patient may be considered the greatest limitation in the effective use of PPIs. Although current studies have found numerous PPI-related side effects, the evidence for most of these studies is relatively weak. In the majority of these studies, the associations are of a small magnitude; additionally, some conclusion are inconsistent with other studies and lack a rational explanation of the corresponding mechanisms. Further, few studies reporting the adverse events of PPIs have attempted to balance the corresponding benefits of PPI use with its purported harm. It is important to remain vigilant regarding the potential PPI-related digestive adverse effects. However, in clinical practice, it is important to carefully evaluate and individually manage patients who require PPI therapy to avoid long-term abuse. This may greatly reduce the likelihood of the related adverse reactions observed in these studies.

The large number of human and animal studies summarized in this review support the link between intestinal flora disruption and PPI-related digestive complications, indicating that the gut microbiota might be a promising target for the prevention and treatment of related diseases. Fecal microbiota transplantation, as a method of re-establishing intestinal microecology, can be effective in certain PPI-related digestive diseases, such as FD and CDAD. Alternatively, some probiotics, such as *Lactobacillus*, have also been used to prevent lower gastrointestinal bleeding. However, more probiotics need to be identified and their roles in PPI-related digestive complications need to be explored further. In addition, more *in vivo* and *in vitro* studies are needed to further establish the mechanisms and molecular targets of the gut microbiota in these diseases. Addressing these identified gaps in knowledge should be a priority in future studies.
